# Therapeutic management of acute type A aortic intramural hematoma

**DOI:** 10.1186/s12872-021-02104-4

**Published:** 2021-06-10

**Authors:** Jue Yang, Changjiang Yu, Xin Li, Juntao Kuang, Zerui Chen, Fei Xiao, Tucheng Sun, Miaoxian Fang, Ruixin Fan

**Affiliations:** 1 Department of Cardiac Surgery, Guangdong Cardiovascular Institute, Guangdong Provincial Key Laboratory of South China Structural Heart Disease, Guangdong Provincial People’s Hospital, Guangdong Academy of Medical Sciences, 96 Dongchuan Road, Guangzhou, Guangdong China; 2 Department of Cardiovascular Surgery, Guangzhou First People’ Hospital, Guangzhou, China

**Keywords:** Aortic intramural hematoma, Therapeutic management, Surgical treatment, Conservative treatment

## Abstract

**Objectives:**

The proper therapeutic management for acute type A aortic intramural hematoma (IMH) is still controversial. The purpose of this study was to compare the outcomes following emergency surgery or conservative treatment for patients with this disease.

**Methods:**

From January 2015 to December 2018, 124 consecutive patients were diagnosed with an acute type A aortic IMH and were included in this study. According to our surgical indications, they were divided into two groups: an operation group (OG) and a conservative treatment group (CG).

**Results:**

Of 124 patients, 83 (66.9%) patients accepted emergency surgery and 41 (33.1%) patients accepted strict conservative treatment. There were no differences between these two groups in early mortality and complications. However, the late mortality of patients in the CG was significantly higher than for patients in the OG. A maximum aortic diameter in the ascending aorta and aortic arch ≥ 45 mm and maximum thickness of IMH in the same section ≥ 8 mm were risk factors for IMH related death in patients undergoing conservative treatment.

**Conclusions:**

The mortality associated with emergency surgery for patients with acute type A aortic IMH was satisfactory. In clinical centers with well-established surgical techniques and postoperative management, emergency surgical treatment may provide a better outcome than medical treatment for patients with acute type A aortic IMH.

## Introduction

Acute aortic intramural hematoma (IMH) is an entity within the spectrum of acute aortic syndrome (AAS), in which a hematoma develops in the media of the aortic wall in the absence of a false lumen and intimal tear [1]. The natural history of acute aortic IMH is highly variable, including hematoma resolution, progression to aortic dissection, aneurysm formation, and aortic rupture [2].

The proper therapeutic management for acute aortic IMH is still controversial, especially for type A aortic IMH. In Western countries, the management of acute type A aortic IMH is emergency or urgent surgery. However, in Japan and Korea, there has been a growing trend towards conservative treatment without surgery for acute type A aortic IMH [2–7]. Thus, we need additional long-term and large scale research to make optimal therapeutic management decisions for these patients. In this report, we retrospectively reviewed our experience in the treatment of acute type A IMH and compared the outcomes for emergency or urgent surgery with conservative treatment for patients with this disease.

## Patients and methods

### Study patients

From January 2015 to December 2018, 124 consecutive patients were diagnosed with acute type A aortic IMH in our hospital. In our study, aortic IMH was defined as the presence of a circular or crescent shaped thickening of the aortic wall (≥ 5 mm) in the absence of detectable blood flow inside(a high attenuation area not enhanced after contrast medium administration) on multidetector computed tomography(MDCT), and accompanied by a clinical presentation compatible with acute aortic syndrome. The acute phase refers to 14 days from onset of this disease. The study was approved by the research ethics committee of Guangdong Provincial People’s Hospital and informed consent was obtained from all participants.

### Management and follow-up

In our emergency room, all patients with suspected AAS received a contrast MDCT and transthoracic echocardiography. The MDCT images were used to confirm a diagnosis of acute type A aortic IMH. The aortic IMH lesion type was classified based on the initial diagnosis (type A or type B) using the earliest imaging data from MDCT and the initial diagnosis was not changed even if the lesion type changed (from type B to type A).

Following a comprehensive assessment, patients with shock (requiring vasoactive drugs), severe hypoxemia (requiring tracheal intubation), moderate or massive pericardial effusion, severe aortic valve regurgitation (AR), acute myocardial infarction and other severe malperfusion syndrome were selected for emergency surgery. Emergency surgery was also performed on patients if the maximum aortic diameter in the ascending aorta and aortic arch was more than 50 mm or the maximum thickness of IMH in the same section was more than 11 mm. Other patients received conservative treatment. They were initially treated with an intravenously administered of beta-blocker, calcium antagonist and pain relieving drug to maintain systolic blood pressure to less than 120 mmHg. Three days later, if these patients were in stable condition, they would receive these medications orally.

After discharge, all patients received strict followed up at one month, three months, six months, 12 months and then yearly. This included a contrast MDCT and transthoracic echocardiography at each follow-up visit. If any patients who received conservative treatment initially were found to meet the above surgical indications at follow-up, emergency surgery was performed.

Patients in the surgical and conservative treatment groups were compared in terms of in-hospital mortality, morbidity, and long-term outcomes.

### Surgical procedure

Usually, cardiopulmonary bypass was implemented via right axillary arterial cannulation. If the right axillary artery was anomalous or too small, the arterial cannulation was performed through the aortic arch with the help of transesophageal echocardiography. Two venous cannulas were respectively inserted into the superior vena cava and inferior vena cava. All patients were cooled to 24 degree centigrade(rectal temperature) and antegrade selective cerebral perfusion was performed through the right axillary arterial cannulation or left carotid arterial cannulation. All patients received ascending aortic replacement and total aortic arch replacement with an intraoperative stent inserted into the descending aorta. If aortic valve disease or dilation of the aortic sinus was present, a Bentall procedure was performed at the same time. For some patients with coronary heart disease or other valvular heart disease, coronary artery bypass grafting and cardiac valvular surgery was performed concomitantly.

### Statistical analysis

Statistical analysis was performed using SPSS version 25.0 for Windows (SPSS, Chicago, IL). Continuous variables were expressed as medians, means ± standard deviations, or both. Categorical variables were expressed as frequencies and percentages. Univariate analysis and logistic regression analysis were performed to identify risk factors for death in patients with conservative treatment. For counting variables, we used the *t* test to perform univariate analysis. For categorical variables, we used the χ^2^ test to perform univariate analysis. If the P value was less than 0.1, the variable would be included in the logistic regression analysis. If the P-value in logistic regression analysis was less than 0.05, we considered the variable to be a risk factor for postoperative death. Survival curves were generated via the Kaplan–Meier method with significant differences assessed for time-to-event data using log-rank tests.

## Results

This study included 110 males and 14 females. Mean age was 53.5 ± 13.1 years, ranging from 26 to 79 years. Time from onset of disease to diagnosis was 18.3 ± 6.7 h, ranging from 12 to 36 h. All patients had a history of hypertension and no patients with Marfan syndrome were included. Ten patients had coronary artery disease and seven patients had a history of stroke. A total of 8.1% and 10.5% of the patients had hyperlipidemia and diabetes, respectively. All 124 patients received transthoracic echocardiography and the grades of aortic valve regurgitation were as follows: 11 none, 53 mild, 47 moderate, 13 severe. All patients received contrast MDCT and 22 patients were found to have massive pericardial effusion. In addition, 38 patients exhibited moderate pericardial effusion and 34 patients had small pericardial effusion. Through the MDCT, we also collected imaging data for every patient. The maximum aortic diameter in the ascending aorta and aortic arch was 45.3 ± 6.5 mm, ranging from 35.8 mm to 55.2 mm. The maximum thickness of IMH in the ascending aorta and aortic arch was 7.6 ± 4.1 mm, ranging from 5.0 mm to 15.4 mm. Before our treatment, nine patients suffered from shock and needed vasoactive drugs, while six patients had severe hypoxemia and underwent tracheal intubation. The incidences of acute myocardial infarction and acute renal failure were 3 (2.4%) and 5 (4.0%), respectively.

According to our indications for emergency surgery, in all 124 patients, 83 (66.9%) patients accepted emergency surgery and 41 (33.1%) patients accepted strict conservative treatment. These patients were divided into two groups: the operation group (OG) and the conservative treatment group (CG). There were no differences between these two groups in terms of age, gender, time between onset and diagnosis, coronary artery disease, diabetes, hyperlipidemia or history of stroke (Table 1). All patients in the OG received ascending aortic replacement and total aortic arch replacement with an intraoperative stent inserted into the descending aorta. Ten also received a Bentall procedure. Two patients received coronary artery bypass grafting due to coronary artery disease. Two other patients received mitral valve repair due to severe mitral valve regurgitation.

**Table 1:** Clinical characteristics of patients

	OG(n = 83)	CG(n = 41)	P value
Age (y)	54.7 ± 14.2	51.1 ± 11.5	1.000
Gender (male/female)	73/10	37/4	0.938
Time between onset and diagnosis (h)	17.7 ± 6.3	19.4 ± 7.0	1.000
Coronary artery disease	6	4	0.892
Diabetes	8	5	0.900
Hyperlipidemia	7	3	1.000
History of stroke	5	2	1.000

* OG*  operation group, *CG* conservative treatment group

### Early outcomes

In the operation group, six patients died before hospital discharge. The early mortality was 7.2% (6/83). The causes of death included heart failure (one patient), cerebral hemorrhage (one patient), sepsis (two patients) and multiple organ dysfunction syndrome (two patients). The incidence of postoperative heart failure, cerebrovascular event, paraplegia, pneumonia, multiple organ dysfunction syndrome and renal failure were 3 (3.6%), 5 (6.0%), 2(2.4%), 4 (4.8%), 2 (2.4%) and 3 (3.6%) patients, respectively. In the conservative treatment group, three patients died before hospital discharge. The early mortality was 7.3% (3/41). The causes of death included cardiac tamponade (two patients) and acute myocardial infarction (one patient). Otherwise, two patients suffered a cerebrovascular event and two other patients had pneumonia. No significant differences between two groups in terms of early outcomes were identified (Table 2).

**Table 2:** Hospital mortality and morbidity

	OG (n = 83)	CG (n = 41)	P value
Heart failure	3	0	0.550
Cerebrovascular event	5	2	1.000
Pneumonia	4	2	1.000
MODS	2	0	1.000
Renal failure	3	0	0.550
Cardiac tamponade	0	2	0.108
Acute myocardial infarction	0	1	0.331
Hospital mortality	6	3	1.000

*OG* operation group, *CG*  conservative treatment group, *MODS*  multiple organ dysfunction syndrome

### Late outcomes

**Fig. 1:**
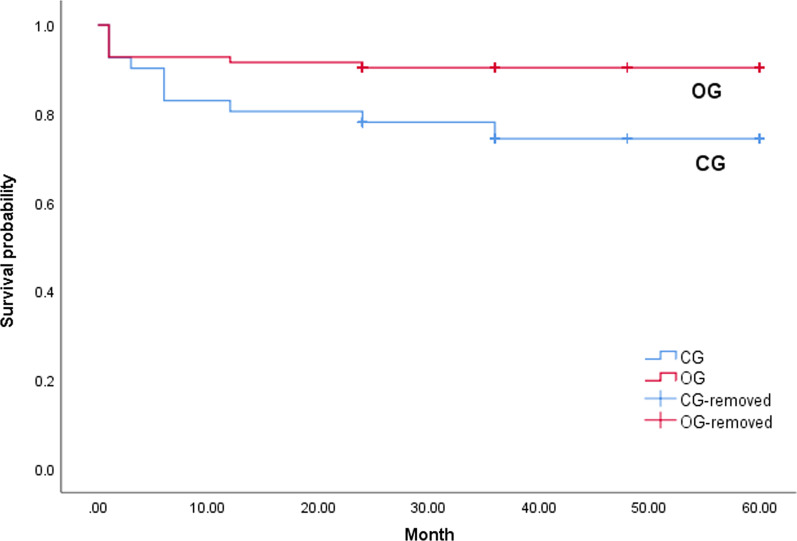
Actuarial survival curves. There was significant difference in actuarial survival at 5 years, which was 90.4% in the OG and 74.3% in the CG (P = 0.010)

After discharge, all surviving patients underwent follow-up at one month, three months, six months, 12 months and yearly. The mean follow-up time was 3.3 ± 1.1 years, ranging from 2 to 5 years. In the operation group, one patient died from a descending aortic rupture one year later and another patient died in a traffic accident. The total IMH related mortality was 8.5% (7/82) due to one censored data. In the conservative treatment group, four patients died of thoracic aortic rupture, two patients died of cardiac tamponade and one patient died of lymphoma. Otherwise, during follow-up, two patients in the CG were found to have dilation of the ascending aorta and in both cases, their maximum ascending aortic diameter was more than 50 mm. They both underwent emergency surgery. The total IMH related mortality was 22.5% (9/40) due to one censored data. There was a significant difference between the two groups in total IMH related mortality (P = 0.032). There was also a significant difference in actuarial survival at 5 years, which was 90.4% in the OG and 74.3% in the CG (Fig. 1).

The mortality of patients in the CG was significantly higher than patients in the OG. Therefore, univariate analysis and logistic regression analysis were performed to identify risk factors for IMH related death in patients with conservative treatment. At first, we chose age, maximum aortic diameter in the ascending aorta and aortic arch, maximum thickness of IMH in the ascending aorta and aortic arch, aortic valve regurgitation, pericardial effusion, coronary artery disease and diabetes as proper risk factors related to death and performed univariate analysis (χ^2^ test) one-by-one. The results of univariate analysis are shown in Table 3.

**Table 3:** Results of univariate analysis

	Death/totality	P value
Age		0.490
≥ 60 y	2/15	
< 60 y	7/25	
Maximum aortic diameter^a^		0.000
≥ 45 mm	6/8	
< 45 mm	3/32	
Maximum thickness of aortic IMH^a^		0.049
≥ 8 mm	5/10	
< 8 mm	4/30	
Aortic valve regurgitation		0.284
Moderate	5/14	
None and mild	4/26	
Pericardial effusion		0.238
Small	7/22	
None	2/18	
Coronary artery disease		1.000
Yes	1/4	
No	8/36	
Diabetes		0.668
Yes	2/5	
No	7/35	

^a^In the ascending aorta and aortic arch

Abbreviations: IMH = intramural hematoma

On the basis of univariate analysis, we singled out maximum aortic diameter in the ascending aorta and aortic arch and maximum thickness of IMH in the same section to undertake binary logistic regression analysis. The results of binary logistic regression analysis are shown in Table 4.

**Table 4:** The results of binary logistic regression analysis

	Death/totality	Odds ratio (OR)	95% Confidence Interval (CI)		P
			Lower limit	Upper limit	
Maximum aortic diameter^a^		15.219	6.239	37.126	0.000
≥ 45 mm	6/8				
< 45 mm	3/32				
Maximum thickness of aortic IMH^a^		23.408	10.618	51.603	0.000
≥ 8 mm	5/10				
< 8 mm	4/30				

a: in the ascending aorta and aortic arch

Abbreviations: IMH = intramural hematoma

On the basis of binary logistic regression analysis, we found that maximum aortic diameter in the ascending aorta and aortic arch and maximum thickness of IMH in the same section were significantly associated with IMH related death in patients with conservative treatment. In the CG, the IMH related mortality of patients with maximum aortic diameter in the ascending aorta and aortic arch ≥ 45 mm or maximum thickness of IMH in the same section ≥ 8 mm was significantly higher than that of other patients. They were risk factors for IMH related death in patients with conservative treatment.

## Discussion

The natural history of acute type A aortic intramural hematoma is extremely variable and controversy still exists regarding whether emergency surgery should be carried out for all acute type A aortic IMH [2–7]. In the European (ESC-AD 2014 guidelines) and American (ACCF-AD 2010 guidelines) guidelines, emergency surgery is recommended for all acute type A aortic IMH [1, 8]. In the Asia Task Force practice guidelines for aortic disease (JCS-AD 2011 guidelines), conservative treatment is recommended for an acute type A aortic IMH in patients with a hematoma thickness < 11 mm and an aortic diameter < 50 mm [9]. However, the data supporting the Asian guidelines mainly came from studies performed in Japan and South Korea and data from the Chinese population is lacking. Moreover, studies supporting both the Eastern and Western guidelines were not randomized trials, and the numbers of enrolled patients were fewer than one hundred even in multicenter studies [6, 10–13]. These numbers are actually too low to provide sufficient statistical power. The data presented in this study may greatly contribute to improvements in practice guidelines for acute type A aortic IMH.

In the present study, our surgical indications for patients with acute type A aortic IMH were based on JCS-AD 2011 guidelines but also included moderate or massive pericardial effusion and severe aortic valve regurgitation into indications of emergency surgery per our clinical experience. Even though our indications for medical treatment were stricter than those in the JCS-AD 2011 guidelines, the mortality of patients with only medical treatment was significantly higher in follow-up than patients treated with surgery. For patients with acute type A aortic IMH, even though contrast MDCT or transthoracic echocardiography on admission or in follow-up did not show any false lumen or intimal tear, blood oozing may have developed gradually and the firmness of the aortic wall may have weakened, allowing cardiac tamponade and aortic rupture to happen. Four patients who underwent medical treatment died of cardiac tamponade and their CT and echocardiography only showed a small pericardial effusion. Four other patients in the conservative group died from an aortic rupture. However, in their contrast MDCT, all had a maximum aortic diameter in the ascending aorta and aortic arch of less than 50 mm and the maximum thickness of IMH in the same section was less than 11 mm. On the other hand, the mortality associated with surgery in acute type A aortic IMH has rapidly decreased due to improvements in surgical techniques and postoperative management. In our study, the total IMH related mortality was only 8.5% yet 10 years ago, this rate ranged from 12.5 to 39.1% [6, 14]. Therefore, nowadays, even when contrast MDCT and transthoracic echocardiography reveal a type A aortic IMH, performing surgery as soon as possible is considered to be a main factor to improving prognosis. Mitsumasa and colleagues [15] have also reported poor outcomes following medical treatment of acute type A aortic IMH. In their research, the freedom from aortic event was only 46.8% at 10 years. They thought emergency surgical treatment may provide a better outcome than medical treatment. In our study, patients in the CG had “much better aorta” than patients in the OG. However, they showed very bad outcomes in terms of aortic events. In the CG, nine patients died of aortic events. In the OG, only one patients suffered from descending rupture. We thought for most patients with type A aortic IMH emergency surgery may be the best choice.

Song and colleagues [6] have reported that in patients of type A aortic IMH with medical treatment, initial aorta diameter and hematoma thickness were independent predictors for adverse clinical events including death and delayed surgery. In our study, we also found that the maximum aortic diameter in the ascending aorta and aortic arch and the maximum thickness of IMH in the same section were risk factors for IMH related death in patients with conservative treatment. In JCS-AD 2011 guidelines, the best cutoff values were 50 mm in aortic diameter and 11 mm in hematoma thickness. However, in our study, the IMH related mortality of patients with maximum aortic diameter in the ascending aorta and aortic arch ≥ 45 mm or maximum thickness of IMH in the same section ≥ 8 mm was significantly higher than that of other patients. Thus, we thought we should make more stringent indications of medical treatment in patients with acute type A aortic IMH.

Finally, in the CG, 30 patients survived. In the follow-up period, 4 suffered complications of pneumonia and heart failure. During the follow-up for the OG, no patient showed these complications. This may be another advantage of surgical treatment. In the CG, bed rest is necessary and important for antihypertensive therapy, particularly in critically sick patients. However, it is associated with risk factors of depression, pneumonia and heart failure [16]. On the other hand, in the OG, the patients could eat and drink on postoperative days. Furthermore, they could take a walk and even work 3 months later because the lesion in the ascending aorta and aortic arch had already been repaired. Therefore, emergency surgery could enable much easier management of patients post-surgery and improve their quality of life compared with medical management.

Our study has several limitations, including all of the limitations related to a retrospective study from a single institution. Furthermore, the small number of patients and the short follow-up time limit the statistical power of the study. More refined studies are necessary in the future to completely resolve the controversy as to the best treatment of acute type A aortic IMH.

## Conclusions

The mortality of emergency surgery for patients with acute type A aortic IMH was satisfactory. In clinical centers with well-established surgical techniques and postoperative management, emergency surgical treatment may provide a better outcome than medical treatment for patients with acute type A aortic IMH.

## Data Availability

The datasets used and/or analysed during the current study available from the corresponding author on reasonable request.

## Abbreviations

CG: Conservative treatment group
IMH: Intramural hematoma
MODS: Multiple organ dysfunction syndrome
OG: Operation group
